# Perspectives of Family Medicine Providers on Nutrition of Maternal-Infant by Group Care Visits: A Cross-Sectional Study

**DOI:** 10.7759/cureus.61428

**Published:** 2024-05-31

**Authors:** Rafeef Alsikhan, Abdulrahman Almotiry

**Affiliations:** 1 Family Medicine, Academy of Family Medicine, Qassim Health Cluster, Al Qassim, SAU

**Keywords:** nutrition, infant, maternal, physicians, family medicine

## Abstract

Background: Group care in child welfare and primary care settings has evolved, becoming a popular approach for maternal and infant health care. This study focuses on the perspectives of family medicine providers on group care visits for maternal and infant nutrition, a crucial aspect of primary healthcare. Hence, this study aimed to explore current practices and opinions regarding the efficacy of group care models in delivering nutrition education to mother-infant dyads.

Methodology: A quantitative, cross-sectional study was conducted among family physicians in Buraydah, Saudi Arabia, from June to August 2023. Participants were recruited using a randomized sampling method from primary healthcare centers. Data were collected through a well-structured, self-administered questionnaire. The total participant count was 60. Statistical analyses were conducted using descriptive and inferential methods.

Results: The majority of participants were men (n=32, 53.3%), under 30 years of age (n=31, 51.7%), and had 0 to five years of experience in medical practice (n=32, 53.4%). A high weekly volume of infant and maternal clinic visits was reported (n=44, 73.3%) but predominantly conducted individual nutrition education sessions (n=60, 100%). A significant majority (n=41, 68.3%) expressed a positive potential for group care in nutrition education.

Conclusion: The study revealed a positive inclination among family medicine providers towards group care models for maternal and infant nutrition education. However, current practices largely involved one-on-one sessions, indicating a gap between the recognition and implementation of group care models. It underscores the need for enhanced integration of group care approaches into clinical practice, highlighting their perceived benefits in efficiency and comprehensiveness. Future steps include implementing group care programs addressing participant concerns and assessing their efficacy in educating mothers on infant nutrition.

## Introduction

There is a long and discussed history of group care in child welfare practice [1]. It is becoming a more common way for women and healthy babies to get primary care [2]. In group care, the primary care provider, and a co-facilitator, such as a social worker, use a guide to make sure that group-driven discussions cover the age-appropriate anticipatory guidance topics after mothers with newborns have one-on-one assessments [3], which is an example of how programs are gradually learning to harness the power of women working together to improve their health and the health of their children [4].

Group care is in place of separate postpartum and well-baby visits (WBVs). The average well-baby visit lasts about seven times, and compliance rates are the highest of any life stage (83.2%) [5]. Additionally, patients pay close attention to the medical advice that their providers give them [6]. Centering parenting, a well-known postpartum group care program, is currently offered in 98 clinics across the United States (US), and significant growth initiatives are in place [7]. According to preliminary studies, 75% to 95% of centering parenting participants are retained [8]. 

Group care addresses several issues with the conventional primary care system. One of these problems is the short duration of one-on-one visits, just 42 seconds of WBVs are dedicated to newborn feeding and the paucity of continued maternal care, where mothers often receive only one visit after giving birth [9, 10]. Also, group care encourages mothers to take an active role in their care, which has been linked to better health outcomes [11]. This method works well in a family medicine clinic because both mothers and infants might be clients. However, not much is known about the potential of group care to provide nutrition education to mother-infant pairs [12].

Educational interventions are widely confessed as efficient in promoting public health strategy, and those aimed at improving complementary feeding practices provide information about proper complementary feeding practices to caregivers of infants/children [13]. Increasing the quality of mother-child interactions through educational interventions has been shown in multiple studies to increase children's cognitive development all around the globe [14]. Nutrition education lowers the number of pregnant women and babies who are overweight by helping moms choose healthier foods and teaching them how to feed their babies well [15]. Machuca et al. found that babies whose mothers got group care, which included nutrition education, were less likely to be overweight or obese at the age of two years than babies whose mothers got traditional care [3]. An appropriate nutrition education program can change the attitudes and beliefs of caregivers and child-feeding practices by improving child growth [16].

A few nutrition education programs focused on both the nutrition of mothers and babies and most of those did it in separate classes for mothers and babies, which has not been found as effective as group care in improving the health of moms and babies [17, 18]. This is probably because mothers were unable to focus on their own needs due to preoccupation with prioritizing the needs of their infants [19]. It is substantially important to understand how family medicine primary care professionals (FPs) interpret group care before introducing it in family practice to discover criteria that would predict the likelihood of implementation success [20]. Due to the lack of studies in group care visits for maternal and infant education and the defect in several researches that explore the views of physicians in general and family physicians in particular in nutrition education, this study aimed to investigate FPs' present methods for teaching about maternal and infant nutrition as well as their opinions on using a group care paradigm to teach the mother-infant dyad about nutrition.

## Materials and methods

Study design and setting

A quantitative cross-sectional study was conducted at primary healthcare centers (PHCCs) in Buraydah from June to August 2023.

Study sampling technique

A purposive convenience sampling method was employed. All participants agreed to participate in the study and based on the inclusion criteria were included.

Inclusion and exclusion criteria

The study included all the FPs who regularly see infants during the WBVs in Buraydah, Al Qassim region of Saudi Arabia. The study also included all clinics from which FPs are part of the family medicine centers in Buraydah, Al Qassim. Also, individuals who agreed to participate in the study, while incomplete questionnaires were not entertained and individuals who refused to participate in the study were excluded.

Sample size

The study population consisted of all FPs who attended PHCCs in Buraydah City during the study period. The total number was estimated to be 60. All FPs involved in WBVs in all PHCs in Buraydah City were included in the study because they represent the majority of PHCCs in the Al Qassim region.C

Data collection

The data were collected by using a well-structured self-administered questionnaire over an average period of one month. The questionnaire was distributed among FPs working at PHCCs belonging to the Ministry of Health (MOH) in the city of Buraydah, Saudi Arabia. The questionnaire was adopted depending on the literature review of the previous studies [14-17]. The questionnaire consists of three parts. Part one was related to the sociodemographic characteristics of the participants including age, gender, qualification, years in medical practice, and number of infants or mothers seen in the clinic per visit. The second part was related to the recent practices while the third part covers the sharing ideas of the FPs. The parameters addressed during the nutrition education communication (NEC) module were regarding environmental health, basics of good personal hygiene, availability of clean and pure drinking water, knowledge about the effective immunization programs, appropriate use of child developmental and nutrition charts, daily consumption of food items from all food groups of the food pyramid, appropriate meal preparation, and foods required for increased lactation and pregnancy.

Statistical analysis

A comprehensive statistical analysis was conducted on the dataset, encompassing both descriptive and inferential methodologies. Firstly, a descriptive analysis was conducted to summarize the sociodemographic characteristics of the participants. Secondly, certain questions were addressed to find out the perspectives of family physicians concerning the nutritional status of mothers and infants during their visits to primary healthcare centers. Chi-square tests/Fisher’s exact tests were utilized to explore potential significant associations between participants’ opinions regarding the impact of group care visits for maternal and infant nutrition education and sociodemographic variables. [AS1] Statistical significance was established at a p-value of 0.05 or lower and a 95% Confidence Interval. All statistical analyses were executed using SPSS version 27.0.0 (IBM Corp., Armonk, NY, USA).

Ethical considerations

Ethical approval of the study was obtained from the Regional Research Ethics Committee via reference number 607-44-17550 on Wednesday, June 21, 2023. No major ethical issues were involved in the current study. Informed consent was taken from every participant. After ethical committee approval, data collection was started. Privacy and confidentiality of the individual information was maintained.

## Results

All participants were family physicians who gave their consent to participate in the study. The majority were men (n=32, 53.3%), aged under 30 years (n=31, 51.7%), and had 0 to five years of experience in medical practice (n=32, 53.4%) (Table 1).

**Table 1:** Descriptive Statistics of Sociodemographic Data of Participants (n=60) The data is presented in frequency (n) and percentage (%).

Variable:		Frequency (Percentage) n (%)
Profession	Family Physician	60 (100.0)
Age (years)	<30	31 (51.7)
	30-45	25 (41.7)
	45-60	4 (6.6)
Gender	Female	28 (46.7)
	Male	32 (53.3)
Years in Medical Practice (years)	0-5	32 (53.4)
	5-10	14 (23.3)
	>10	14 (23.3)

Regarding their recent practices in their clinics, the majority (n=44, 73.3%) reported having five to 10 infants in the clinic each week. Moreover, it was mentioned to have 10-30 women (n=21, 35%) or >35 women (n=11, 18.3%) visiting the clinic each week. It was acknowledged that all nutrition education for mothers and infants sessions were individually conducted (n=60, 100%). A significant portion (n=34, 56.7%) reported that these individual sessions were implemented every single visit (Table 2).

**Table 2:** Descriptive Statistics of Recent Practices (n=60) The data is presented in frequency (n) and percentage (%).

Variable:		Frequency (Percentage) n (%)
Number of infants seen in clinic per week	5-10	44 (73.3)
	10-30	12 (20.0)
	>30	4 (6.7)
Number of mothers seen in clinic per week	5-10	28 (46.7)
	10-30	21 (35.0)
	>30	11 (18.3)
How are the nutrition education sessions typically conducted?	Individually	60 (100.0)
How often do you provide these individual sessions?	Every visit	34 (56.7)
	Every other visit	15 (25.0)
	Rarely	11 (18.3)

**Figure 1:**
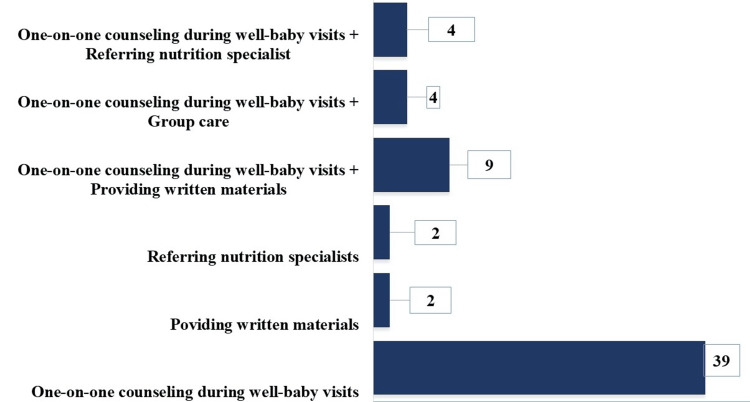
Number of Participants Replied to The Question, How Do You Currently Educate New Moms on Prenatal Nutrition And Infant Feeding?

When participants were asked about their methods to educate new mothers on prenatal nutrition and infant feeding, the majority adopted single while the rest followed mixed approaches. Specifically, 39 out of 60 (65%) mentioned offering one-on-one counseling services during mothers' visits to the clinic. Two (3.33%) participants replied that they exclusively referred mothers to nutrition specialists and another two (3.33%) provided written materials. The remaining participants employed mixed approaches. Added to counseling services, nine (15%) participants mentioned providing written materials, four (6.66%) referred mothers to nutrition specialists, and another four (6.66%) conducted group care sessions (Figure 1).

**Figure 2:**
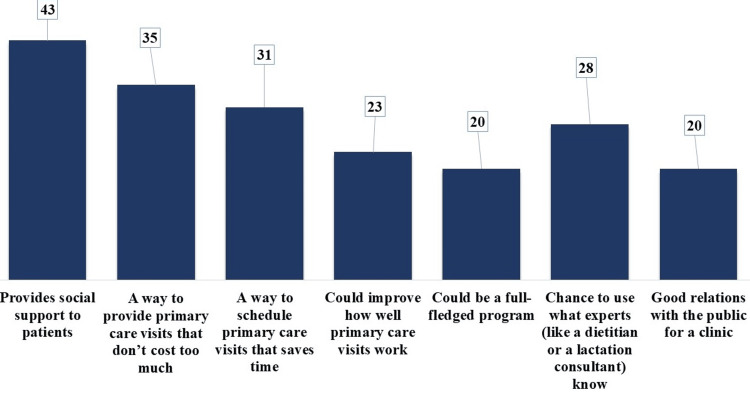
Frequency of Ideas Shared by Participants for Nutrition Education Delivery

Participants expressed their ideas for nutrition education delivery through a multiple-choice question. The frequency of each selected option was counted to find out their most valuable ideas. In descending order, it was considered that nutrition education delivery provides social support to patients (43 times), doesn't cost too much (35 times), saves time (31 times), helps experts to practice their knowledge (28 times), improves the work of primary care visits (23 times), develops better doctor-patient relationship (20 times), and could be a comprehensive program (20 times) (Figure 2).

**Figure 3:**
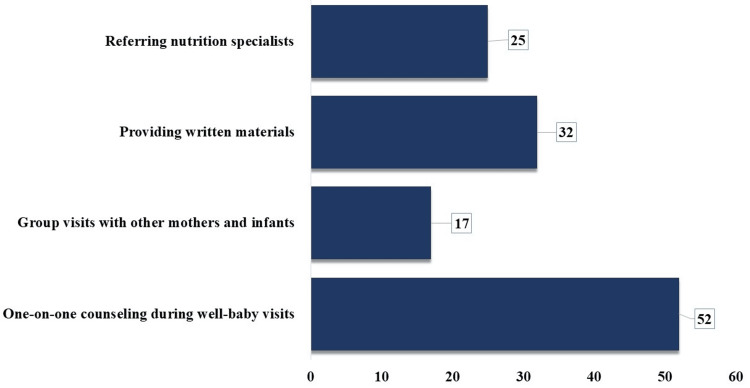
Frequency of Approaches Suggested by Participants to Deliver Mother-Infant Nutrition Education

Participants proposed one-on-one counseling as the most effective approach to delivering mother-infant nutrition education in a family medicine clinic (52 times). Providing written materials (32 times), referring to nutrition specialists (25 times), and conducting group visits (17 times) were also recommended (Figure 3).

A significant majority of participants (n=41, 68.3%) expressed a positive potential for group care in educating mothers on nutrition. Most of them considered themselves knowledgeable about maternal and infant nutrition to varying degrees (n=47, 78.4%). Additionally, the majority (n=38, 63.3%) acknowledged noticing an impact of maternal and infant education on nutrition levels in their patients. The primary impacts reported were improving health and immunity (n=12, 48%), changing mothers' practices (n=7, 28%), achieving a better quality of life (n=2, 8%), motivating mothers (n=2, 8%), and better weight control (n=2, 8%) (Table 3).

**Table 3:** Descriptive Statistics of Ideas Shared by Participants (n=60) The data is presented in frequency (n) and percentage (%).

Variable:		Frequency (Percentage) n (%)
How do you see the potential of group care for educating mother-infant pairs on nutrition?	Neutral	19 (31.7)
	Somewhat positive	23 (38.3)
	Very positive	18 (30.0)
How would you rate your level of knowledge on maternal and infant nutrition?	Neutral	11 (18.3)
	Somewhat knowledgeable	41 (68.4)
	Very Knowledgeable	6 (10.0)
	Very uninformed	2 (3.3)
Have you noticed any impact of maternal and infant education on the nutrition level of your patients?	Yes	38 (63.3)
	No	22 (36.7)
If yes, describe the impact you have observed (n=25)	Change Practice of Mother	7 (28.0)
	Good health & immunity	12 (48.0)
	Happy, good quality of life	2 (8.0)
	Motivation of mothers	2 (8.0)
	Weight control	2 (8.0)

Factors influencing participants' perspectives toward the impact of group care visits for maternal and infant nutrition education were further investigated. None of the sociodemographic variables or variables related to recent practices were found to be associated with their perspectives on nutrition education. However, their reported knowledge level on maternal and infant nutrition showed a significant association (p-value = 0.004). Participants who self-reported as knowledgeable had more positive perspectives (n=35, 85.3%) compared to those who were neutral or uninformed (n=6, 31.5%) (Table 4).

**Table 4:** Factors Associated with Participants’ Opinion Regarding the Impact of Group Care Visits for Maternal and Infant Nutrition Education (n=60) The data is presented in frequency (n) and percentage (%). a: Chi-square test, b: Fisher’s exact test

Variable:		Neutral (n=19)	Positive (n=41)	p-value
Age (years)	<30	10	21	0.428^b^
	30-45	9	16	
	45-60	0	4	
Gender	Female	8	20	0.630^a^
	Male	11	21	
Years in Medical Practice (years)	0-5	11	21	1.000^b^
	5-10	4	10	
	>10	4	10	
Number of infants seen in clinic per week	5-10	12	32	0.450^b^
	10-30	5	7	
	>30	2	2	
Number of mothers seen in clinic per week	5-10	6	22	0.270^b^
	10-30	8	13	
	>30	5	6	
How often do you provide these individual sessions?	Every visit	12	22	0.170^b^
	Every other visit	2	13	
	Rarely	5	6	
How would you rate your level of knowledge on maternal and infant nutrition?	Neutral/ uninformed	7	6	0.004^b^
	knowledgable	12	35	
Have you noticed any impact of maternal and infant education on the nutrition level of your patients?	Yes	12	26	1.000^a^
	No	7	15	

## Discussion

This study aimed to explore the perspectives of family medicine providers on maternal and infant nutrition education within group care settings. The current study findings revealed a significant inclination towards the potential benefits of group care in nutrition education for mothers and infants, aligning with existing literature emphasizing the efficacy of group-based approaches in healthcare. The majority of the family physicians in the current study were under 30 years old (n=31, 51.7%) with 0 to five years of medical practice experience. This younger, relatively less experienced demographic could be more open to innovative care models like group visits, as suggested by studies indicating that younger healthcare professionals are often more adaptable to new methods and ideas [21]. In addition, the slight male predominance (n=32, 53.3%) in the current study contrasts with some literature indicating an increasing feminization of family medicine [13]. However, it aligns with the traditional gender distribution in certain regions [22].

The high number of infants and mothers seen weekly by family physicians highlighted the potential reach and impact of group care sessions. Given that each session accommodates multiple patients, group care can be a more efficient approach, as supported by efficiency studies in group care models [23]. Participants' prior experience with group care in their clinic or another hospital system certainly influenced their preference for this method of delivering nutrition training to mother-infant dyads. Although concerns were voiced, the good experiences of the participants enhanced the potential success of group care sessions. In the initial evaluations of centering parenting, the participants listed several benefits and drawbacks of group care. The efficiency of providing social assistance and anticipatory counseling was frequently mentioned as an advantage, but difficulties with recruiting and implementation logistics were often cited as challenges. The capacity of group care to provide social assistance is remarkable due to its correlation with favorable postpartum results, such as extended breastfeeding. Moreover, the fact that all nutrition education sessions are currently conducted individually points to a significant opportunity for introducing group care models. This is particularly relevant to a study that emphasizes the benefits of group learning environments, such as enhanced peer support and shared learning [24].

The reliance on one-on-one counseling (n=39, 65%) for educating new mothers on prenatal nutrition and infant feeding, while effective, might not be as efficient or comprehensive as group sessions. Some studies reported that a supportive and interactive environment can be achieved through group sessions, resulting in better retention of information. Some physicians prefer using mixed approaches, which results in the willingness to better adapt and customize education methods. This flexibility is key to addressing diverse patient needs, as highlighted in studies on patient-centered care [25]. The recognition of social support and cost-effectiveness as major benefits of group care aligns with literature emphasizing the importance of social interaction in healthcare and the economic benefits of group-based interventions [12, 26]. The perception of group care as time-saving and offering a comprehensive program resonates with studies indicating that group care can be more efficient and provide holistic education compared to traditional one-on-one sessions [27].

A substantial portion of participants (n=41, 68.3%) see positive potential in group care for nutrition education is a promising indication of its acceptability. This aligns with the growing body of evidence supporting the effectiveness of group care in various healthcare settings [8]. The majority of family physicians consider themselves knowledgeable about maternal and infant nutrition and observing impacts on nutrition levels in their patients underscores the importance of provider expertise in effective education. This is consistent with studies linking provider knowledge to better patient outcomes [28]. The reported impacts on health, immunity, and mother practices are significant, supporting literature that emphasizes the long-term benefits of effective nutrition education in early life stages [8].

The absence of significant associations between physicians’ perspectives on group care and their sociodemographic characteristics suggests that attitudes towards group care might be influenced more by other factors, such as personal beliefs or institutional culture, rather than age or gender. The significant association between self-reported knowledge and positive perspectives toward group care suggested that enhancing physician education in nutrition and group care methodologies could be crucial for its broader adoption and success [28].

The current study findings indicated a clear recognition among family medicine providers of the potential benefits of group care for maternal and infant nutrition education. However, there is a notable discrepancy between this recognition and current practice, which predominantly involves individual sessions. This gap presents a significant opportunity for healthcare systems to innovate and restructure traditional practices. The positive inclination towards group care, especially among younger and less experienced physicians, could be leveraged to drive change in healthcare delivery models [29]. Given the high volume of patients seen by family physicians, as indicated in the current study, transitioning to group care models could substantially increase the efficiency and reach of nutrition education programs. Furthermore, the emphasis on social support, cost-effectiveness, and comprehensive care in group settings resonates with current healthcare trends favoring patient-centered and economically sustainable models. The significant association between self-reported knowledge and positive perspectives towards group care underlines the need for targeted education and training for healthcare providers in this domain [20].

The current study exhibited some key strengths. One of the primary strengths of the study was its focused approach, targeting a specific and relatively unexplored aspect of healthcare. The qualitative methodology employed allows for an in-depth exploration of the perspectives, beliefs, and attitudes of family medicine providers, offering a nuanced understanding that quantitative methods might overlook. Additionally, the cross-sectional design provides a snapshot of current opinions and practices, which is essential for identifying contemporary trends and needs in healthcare settings. However, the study had several limitations too. The findings might not be generalized to other areas with distinct economic, cultural, or healthcare dynamics due to the geographical limitations of Buraydah, Saudi Arabia. Although the study's cross-sectional nature helped in collecting the present viewpoints, it is not appropriate for establishing causation or monitoring changes over time. In the case of self-reported data, there is a chance of biases such as social desirability bias, whereby participants might provide answers they thought would be favorable to others. The sample size, although reasonable, might not fully capture the breadth of opinions and practices in the wider field of family medicine.

Addressing these limitations in future research could involve expanding the geographical scope of the study, incorporating quantitative elements for a mixed-methods approach, and implementing longitudinal designs to observe changes over time. Strategies to minimize self-report biases and enhance response rates would also contribute to the robustness and reliability of future findings [27]. As a result of the current study, family medicine primary care providers might accept group care models. However, since this study focused on Buraydah’s family medicine provider, it is recommended that a larger-scale study should be conducted in all regions of Saudi Arabia. Also, the challenges or barriers that might hinder the delivery of nutrition education must be identified and resolved before implementation. Further, family medicine providers must be encouraged about the efficacy of group care, especially regarding nutritional education.

## Conclusions

This study revealed a positive attitude of family medicine providers towards the potential of group care models. It highlighted a gap between the recognition of these benefits and their practical implementation. The findings suggested a need for enhanced integration of group care approaches in clinical practice, emphasizing their efficiency and comprehensive nature. Important future steps include putting into practice a group care program that addresses the concerns brought up by participants and assessing how well group care educates mothers of infants about nutrition.
